# Livestock-Associated *Staphylococcus aureus*: The United States Experience

**DOI:** 10.1371/journal.ppat.1004564

**Published:** 2015-02-05

**Authors:** Tara C. Smith

**Affiliations:** Department of Biostatistics, Environmental Health Sciences, and Epidemiology, Kent State University, Kent, Ohio, United States of America; Duke University Medical Center, UNITED STATES

## Background and Overview

*Staphylococcus aureus* is a gram-positive bacterium that colonizes a variety of animal species [1]. *S. aureus* infections in animals are most commonly reported as a cause of mastitis in dairy-producing animals (including cattle and goats) and “bumblefoot” in chickens [2], as well as being identified as a pathogen of farmed rabbits [3]. Most reports characterizing animal-associated *S. aureus* have demonstrated that strains affecting animals are distinct from those infecting humans, suggesting that there are host-specific lineages which only rarely cross species boundaries [4].

**Figure 1:**
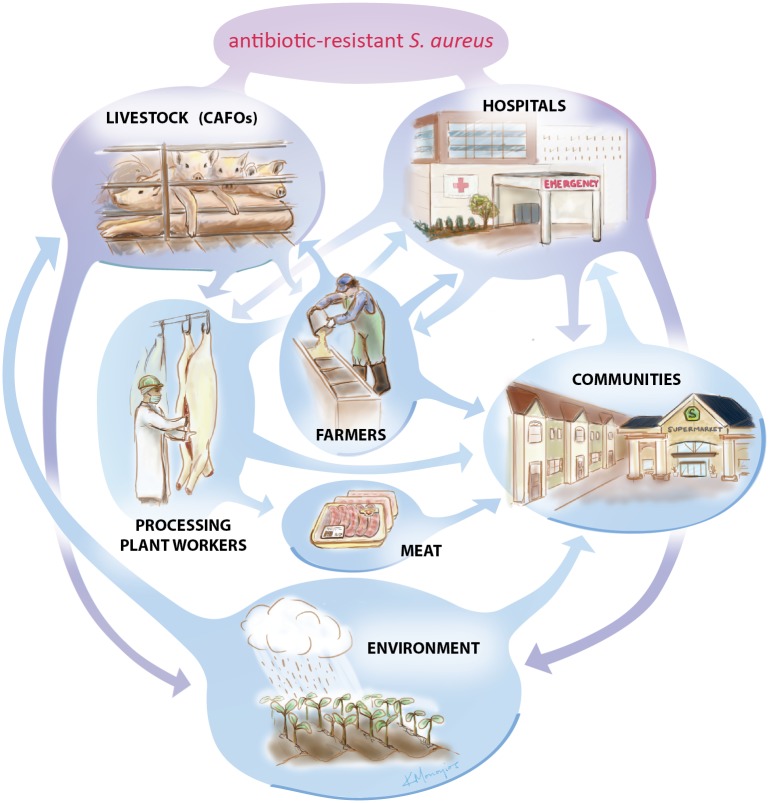
The tangled web of *S. aureus* in the US. Antibiotic-resistant *Staphylococcus aureus* is a growing public health concern, but tracing the origins of the bacterium is complicated. Evidence suggests that antibiotic-resistant strains of *S. aureus* can spread in livestock operations and hospitals where antibiotics are regularly used. These antibiotic-resistant organisms can then spread into communities and the environment. More research is needed to determine exactly how these transfers occur. Image by Kalliopi Monoyios.

Livestock-associated strains may evolve on farms because of the use of antibiotics in animal husbandry. These may be used as feed additives for growth promotion in industrial livestock and poultry [5], for prevention of disease within a herd, or for treatment of an existing disease outbreak. Agricultural-use antibiotics include many classes that are relevant for human health, including tetracyclines, macrolides, penicillins, and sulfonamides, among others. Antimicrobial resistance generated during animal husbandry may then be spread to the general human population in a number of different manners: contact with contaminated meat products (via handling or ingestion); occupational contact (farmers, meat packers, butchers, etc.) and potential secondary spread into the larger community from those who are occupationally exposed; entry into and transmission via hospitals or other health care facilities; or spread via environmental routes including air, water, or manure in areas in proximity to live animal farms or crop farms where manure has been used as a fertilizer (Fig. 1).

While methicillin resistance has been the most commonly investigated phenomenon and will be the main topic of this review, resistance to any of these antibiotics can occur and can potentially be a threat to successful treatment of *S. aureus* infections and therefore to human health outcomes. As such, my research group and others have begun to look more broadly at any *S. aureus* present on farms, including those that may be susceptible to methicillin but resistant to other antibiotics.

## Livestock-Associated Methicillin-Resistant *Staphylococcus aureus* (MRSA): An Overview

In the early part of the 21st century, a novel pig-associated strain of MRSA was identified: sequence type 398 (ST398) and related strains (collectively grouped into clonal complex 398, or CC398, reviewed in [6]). CC398 was first identified in pigs and swine workers but has since been found in other animals (including cattle, poultry, and dogs as well as humans) in a number of countries in Europe, Asia, and North and South America, as well as Australia. The discovery of this strain led to the addition of livestock-associated MRSA (LA-MRSA) to the lexicon, to complement hospital-associated (HA) and community-associated (CA) strains.

In most European countries, CC398 remains the most commonly identified type of LA-MRSA [6–9], leading to a presumption that the terms LA-MRSA and CC398 are practically interchangeable. However, while CC398 strains have been found in livestock across the globe, the epidemiology of livestock-associated *S. aureus* has been found to differ in other geographic areas. Several Asian studies have demonstrated that a different strain of MRSA, ST9, appears to be the prominent type of LA-MRSA in several Asian countries [10–14]. Poultry may harbor CC398 strains [15–17] but also other types unrelated to CC398, including CC5 [15, 18] or other types [17]. In the United States, the diversity of livestock-associated *S. aureus* appears to be higher than that identified in Europe or Asia, with reports of both CC398 as well as a variety of “human” types of *S. aureus* in live animals, as described below.

The epidemiology of CC398 and other strains found in both animals and humans [12] has led to a reexamination of the idea of host specificity in *S. aureus*. CC398 appears to be frequently shared between animals and humans and is capable of causing active symptomatic infections in both species [19, 20]. Furthermore, both CC398 and a poultry-adapted *S. aureus* strains of CCT5 have been phylogenetically analyzed and appear to have originated in humans, who transmitted strains to animals, in which the strains subsequently spread and evolved a variety of host adaptations [21, 22]. As such, there exist both human-associated CC398 strains as well as true livestock strains, complicating studies of origin or host association based only on knowledge of sequence type.

## Epidemiology of CC398 and Other Livestock-Associated *S. aureus* in the US

The epidemiology of LA-SA in the US appears to be notably different than in European countries, where the bulk of LA-SA research has been carried out. While early studies on farms and of meat-identified CC398 strains in animals, farm workers, and meat products, [23, 24], contemporaneous studies also documented CC398 in populations with no obvious livestock contact [25–27]. In one Texas publication carried out in a jail setting rather than on a farm, CC398 isolates made up a significant portion (13.2%) of all methicillin-susceptible *S. aureus* (MSSA) identified within this population. Clearly, the association of CC398 exclusively with an agricultural reservoir did not appear to hold in the US.

While CC398 can have LA as well as human versions, other human strains of *S. aureus* have also been found in US livestock. Studies carried out on swine farms in the US have identified human strains within the noses of live animals [28–30] or as components of environmental samples of farm dust [31]. Several papers have found CC5 strains rather than CC398-associated types to be the dominant strain isolated from pig farms in both Iowa and Ohio [31, 32], while others have found CC398 to be the most common molecular type [23, 33]. Three studies in North Carolina examining workers on pig farms and in processing plants similarly found substantial diversity among *S. aureus* isolated from workers, including CC398, CC5, and CC8 strains, among others [34–36].

## Transmission between Animals and Humans in the Farming Setting

Studies of individuals living in proximity to concentrated animal feeding operations (CAFOs) support the idea that nonlivestock strains may be spreading within areas proximal to farms. Two independent studies carried out in Iowa and Pennsylvania that examined the relationship between animal farms and MRSA found an increased risk of MRSA colonization or infection in those living close to farms or in areas where manure was spread on fields [37, 38]. In both studies, however, no classic LA strains were found when molecular typing was carried out on isolates collected. This suggests that either strains other than LA isolates are evolving on farms (consistent with on-farm sampling described above) or that it may be the presence of antibiotic resistance genes and antibiotic residues on farms that are moving to the subjects’ own bacterial flora and causing a shift toward antibiotic-resistant strains in these populations, or perhaps a combination of both mechanisms. Firm conclusions are difficult to make in the absence of a concerted, national-level on-farm sampling effort, which is difficult to carry out in the US because of private/corporate ownership of many farms and laws in several states that are unfriendly to farm visitors.

## Human Infections with Livestock-Associated *S. aureus* Strains

A number of human infections with CC398 strains have been reported. Most of these have been documented in Europe [39–41]; however, CC398 infections from the US [26, 42, 43] and Canada [44] have been reported as well. Because many infection reports were published prior to the recognition of distinct lineages of CC398, it is not always clear, particularly for individuals lacking exposure to livestock, whether the CC398 strains identified are ancestral human strains, or derived livestock types. This has significance for prevention and treatment, as human-origin strains appear to be more virulent than true livestock strains but may also be less likely to be multidrug resistant (and as such, more easily treatable) [22]. Nonetheless, the majority of reported infections with CC398 strains appear to be similar in scope to community-associated *S. aureus* strains, causing skin and soft tissue infections and, more rarely, serious invasive infections and death.

## Potential for Meat Products as a Source of LA-SA in the Community

Just as a variety of human and livestock strains have been found in live animals on farms, so have they been found in meat products sampled in the US [18, 24, 45–49]. CC398 strains have been found in pork and chicken products in the US and appear to be the dominant contaminating strains in raw turkey meat. *S. aureus* may be transmitted to humans from meat products by handling of contaminated products or by the cross contamination of household surfaces (such as countertops and sinks), which are then touched by family members. While antibiotic use on farms may drive selection of antibiotic-resistant strains of *S. aureus* that eventually end up in meat products, eliminating consumer exposure to such bacteria is not as straightforward as simply purchasing products raised in an antibiotic-free environment. In a study examining conventional versus antibiotic-free pork products, no difference was found in prevalence of MRSA between these types of samples [46]. This was a different result obtained from sampling results on conventional versus antibiotic-free farms [33], suggesting the potential for either contamination of pigs with MRSA in the lairage area prior to slaughter or contamination of meat products during processing or packaging, either via humans in the plants who may spread MRSA to meat products or from bacterial residues present from conventional products. It is currently not known what the risk is to consumers from *S. aureus*–contaminated meat products.

## Conclusions, Significance, and Future Studies

Livestock-associated *S. aureus* is an emerging category of *S. aureus* throughout the world. Currently, the research carried out has focused more closely on carriage than on transmission and infection, but these strains appear to be less likely to cause human infections and to spread person-to-person than typical human strains [50]. However, these conclusions should be noted with caution, as few well-designed prospective studies have been conducted to answer these questions to date.

Recent research suggests that bidirectional transmission of strains of *S. aureus* between humans and livestock is not a rare occurrence. In addition to the movement of CC398 between animals and humans, studies have suggested that a human pandemic clone, CC97, had its origin in cattle [51]. Additionally, antibiotic resistance genes, including *mec*A [52, 53] and *mec*C [54, 55], have been suggested to have an animal origin.

Currently, we are limited in the ways we can distinguish whether any particular strain of *S. aureus* is a human or livestock-adapted isolate. We can use the presence of marker genes, including the loss *scn* and presence of *tet*(M), both of which are genotypes associated with livestock adaptation of CC398 lineages [22, 56] or examine the presence of a single-nucleotide polymorphism (SNP) that has also been identified in this clade [56]. However, large-scale studies validating these markers in other lineages (CC5, CC8, and more) are lacking. Additional large-scale studies in both human and animal populations are necessary in order to gather isolates that are epidemiologically well characterized. These isolates can then be analyzed in order to validate current genomic markers, as well as to identify novel ones in lineages besides CC398.

*S. aureus* surveillance is most commonly carried out within a human clinical or hospital setting, with far fewer research dollars devoted to analysis of carriage within communities, particularly in a rural setting, and very little research examining animal strains. As such, it is likely we are missing other spillover events of *S. aureus* from livestock to humans or vice versa. To track such events and facilitate both surveillance and source tracking of novel isolates, the buy-in of industry is needed. All too often, the relationship between public health and the agricultural and food industry is one of antagonism rather than assistance. Working together will mean both safer food products and well-protected workers. More attention to this type of research is needed, as we are rapidly approaching a “post-antibiotic era” [57]. The effectiveness of antimicrobial stewardship in the clinical setting may be reduced if pathogens and resistance genes from the agricultural environment are repeatedly, but silently, being introduced into the human population [58].
